# Exploring global research trends in Chinese medicine for atherosclerosis: a bibliometric study 2012–2023

**DOI:** 10.3389/fcvm.2024.1400130

**Published:** 2024-06-17

**Authors:** Moye Tan, Jiuyuan Wang, Zhengxin Chen, Xuejiao Xie

**Affiliations:** ^1^ College of Chinese Medicine, Hunan University of Chinese Medicine, Changsha, China; ^2^ College of Chinese Medicine, Guangzhou University of Chinese Medicine, Guangzhou, China

**Keywords:** atherosclerosis, cardiovascular disease, bibliometric analysis, Chinese medicine visual analysis, CiteSpace

## Abstract

**Background:**

While Traditional Chinese Medicine (TCM) boasts an extensive historical lineage and abundant clinical expertise in addressing atherosclerosis, this field is yet to be penetrated adequately by bibliometric studies. This study is envisaged to evaluate the contemporary scenario of TCM in conjunction with atherosclerosis over the preceding decade while also identifying forthcoming research trends and emerging topics via the lens of bibliometric analysis.

**Methods:**

Literature pertaining to TCM and atherosclerosis, circulated between January 1, 2012 and November 14, 2023, was garnered for the purpose of this research. The examination embraced annual publications, primary countries/regions, engaged institutions and authors, scholarly journals, references, and keywords, utilizing analytical tools like Bibliometrix, CiteSpace, ScimagoGraphica, and VOSviewer present in the R package.

**Result:**

This field boasts a total of 1,623 scholarly articles, the majority of which have been contributed by China in this field, with significant contributions stemming from the China Academy of Traditional Chinese Medicine and the Beijing University of Traditional Chinese Medicine. Moreover, this field has received financial support from both the National Natural Science Foundation of China and the National Key Basic Research Development Program. Wang Yong tops the list in terms of publication count, while Xu Hao's articles take the lead for the total number of citations, positioning them at the core of the authors’ collaborative network. The Journal of Ethnopharmacology leads with the most publications and boasts the greatest total number of citations. Principal research foci within the intersection of Chinese Medicine and Atherosclerosis encompass disease characteristics and pathogenic mechanisms, theoretical underpinnings and syndrome-specific treatments in Chinese medicine, potentialities of herbal interventions, and modulation exerted by Chinese medicines on gut microbiota.

**Conclusion:**

This analysis offers a sweeping survey of the contemporary condition, principal foci, and progressive trends in worldwide research related to Traditional Chinese Medicine (TCM) and atherosclerosis. It further delves into an in-depth dissection of prominent countries, research institutions, and scholars that have made noteworthy strides in this discipline. Additionally, the report analyzes the most cited articles, research developments, and hotspots in the field, providing a reference for future research directions for clinical researchers and practitioners.

## 1 Introduction

In tandem with escalating economic advancement and enhanced living standards, cardiovascular disease has ascended to the leading cause of global mortality. Significantly, this disease is not exclusively restricted to middle-aged and elderly demographics, marking the emergence of a rejuvenation trend (1). Cardiovascular disease is often caused by hyperlipidemia, blood viscosity, atherosclerosis, and hypertension (2, 3). Atherosclerosis (AS) is a pivotal contributor to the development of cardiovascular disease, implicated in the formation of atheromatous plaques on arterial walls, which result in arterial stenosis (4). The pathogenesis of AS exhibits complexity and diversity. While inflammation, lipid infiltration, oxidative stress, and endothelial damage have been proposed as primary causative factors, they lack completely explanatory power of the exact pathogenesis (5, 6).

A range of modern therapeutic strategies has garnered substantial attention. Within dietary considerations, heightened consumption of low-salt and animal- or plant-derived foods has been correlated with a diminished risk of atherosclerosis (7). In contrast, Traditional Chinese Medicine (TCM) has a wealth of clinical experience and a deep historical background in the treatment of atherosclerosis. With ongoing research, extensive investigations have been conducted into the mechanisms of action underpinning TCM monomers, specialized Chinese medications, and complex formulations (8). This has culminated in the incremental elucidation of the active constituents and molecular underpinnings of TCM's therapeutic efficacy in combating atherosclerosis (4). Macrophages are pivotal in the pathogenesis of atherosclerosis (9). Notably, one study found that quercetin could attenuate lipid accumulation induced by oxLDL in the RAW264.7 macrophage lineage, thus impeding their senescence (10). Inflammation has been posited as an emergent mechanism and putative therapeutic target in atherosclerosis (11, 12). Furthermore, the Naoxintong (NXT) capsule has been shown to inhibit the expression of inflammatory molecules by lowering the serum levels of TNF-α and P-selectin (13). Zhang et al. unearthed in their experiments involving ApoE^−/−^ mice that Yinxing tongmai Decoction could boost cholesterol efflux by initiating the PPARγ-LXRα-ABCA1/ABCG1 signaling pathway, thus relieving AS (14). However, the complexity of Traditional Chinese Medicine's composition, uncertainty regarding its pharmacological mechanism of action, and the lack of scientific evidence evaluated by critically reviewed clinical trials have resulted in a lack of coherence in understanding and developing TCM treatment for AS (15). Herein lies an urgent imperative to organize and summarily delineate this issue in a systematic and objective manner. These challenges have gravely thwarted the additional progression of Chinese medicine.

Bibliometrics represents a discipline utilizing mathematical and statistical methods to quantitatively evaluate the knowledge vectors across diverse areas of study. Its wide application encompasses the analysis of the evolution potential of research fields, knowledge structure dynamics, collaboration intensity, recognition of research hotspots, and the prediction of development trajectories (16). Its emergence sheds light on the plight of TCM. The bibliometric methodology enables the depiction of collaborative networks among authors and countries, clustering of hotspot distribution, historical citation networks, and prognostication of future development trajectories in the treatment of AS with TCM (17). Therefore, this study selected relevant papers published between 2012 and 2023.

This paper aims to analyze a decade's worth of fundamental research to evaluate the advancement and evolutionary features of this discipline. The study embarks on an extensive examination of the role and impact of TCM in treating atherosclerosis. It aims to equip future investigations in the field of TCM and atherosclerosis with valuable reference data and valuable insights.

## 2 Materials and method

### 2.1 Data collection and filtration

In this study, we used the Web of Science (WOS) core database as the data collection source. The WOS database is highly respected by a wide range of researchers for its comprehensive coverage of a wide range of publications in a variety of subject areas, its accuracy and information richness, and its citation analysis function, which includes citation tracking, citation counts, and H-index; it includes earlier literature than other databases, and it can be searched by authors, affiliations, countries, journals/titles, and a wide range of subject categories. We selected SCI-E to ensure breadth and accuracy of data collection. We used advanced search terms after confirming the search terms, and in the search formula, subject terms were joined with subject terms by the “AND” symbol, and free terms (synonyms of subject terms) were joined with free terms by the “OR” symbol.

**Figure 1:**
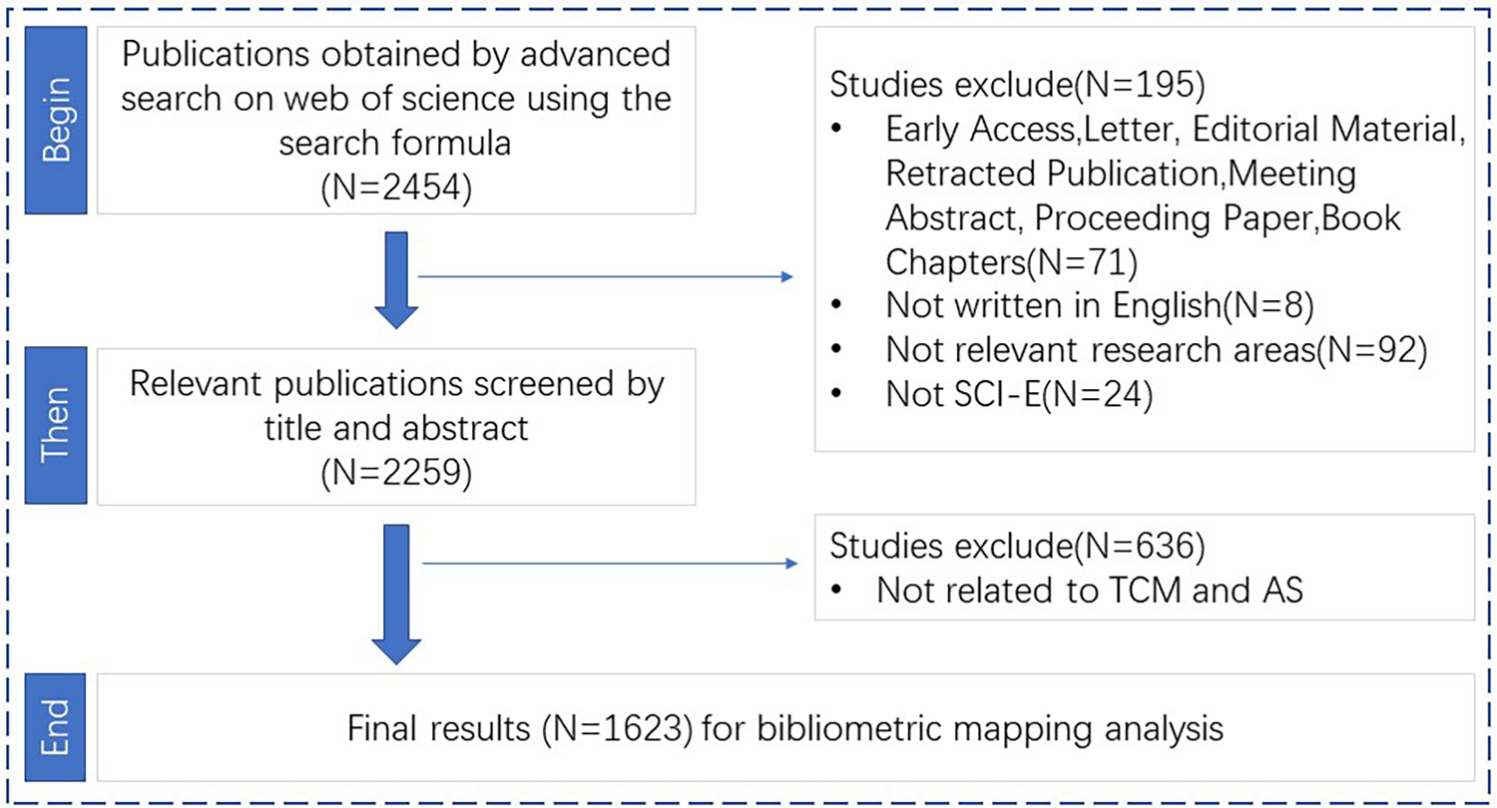
Article search and screening process.

Search #1 first: TS = (“Chinese medicine” OR “Chinese herb” OR “Chinese drug” OR “Chinese herbal medicine” OR “Chinese medicinal herb” OR “Chinese herbal drug” OR “traditional Chinese medicine” OR “TCM” OR “Chinese medicinal plant” OR “Chinese materia medica” OR “Chinese decoction” OR “Chinese formula” OR “Chinese prescription” OR “Chinese patent medicine” OR “Chinese herbal formula” OR “Chinese herbal compound” OR “Chinese herbal prescription” OR “Chinese herbal ingredient” OR “Chinese herbal preparation” OR “herbal medicine” OR “traditional medicine”); #2: TS = (“carotid atherosclerosis” OR “carotid artery stenosis” OR “carotid artery” OR “carotid plaque” OR “atherosclerosis” OR “coronary atherosclerotic heart disease” OR “coronary heart disease” OR “coronary artery disease” OR “ischemic heart disease” OR “angina pectoris” OR “acute coronary syndrome” OR “myocardial infarction”), and then searched for #1 and #2. To ensure the accuracy of the study, we performed all searches and screenings on the same day (November 14) using the same strategy to ensure the timeliness and relevance of the conclusions drawn. In the search results, we first eliminated articles with titles and abstracts that were not relevant to TCM/atherosclerosis research. We then ensured that the inclusion criteria were met by reading the full text. This process was performed by Jiuyuan Wang and Zhengxin Chen, with Moye Tan making the final decision in case of disagreement. In total, the search yielded 2,342 articles, with all additional system parameters for literature search set to their default settings. Upon further scrutiny, we procured a total of 1,623 documents (Figure 1).

### 2.2 Bibliometric analysis

For the data analysis part, we used the bibliometric analysis toolkit in Citespace, VOSviewer, ScimagoGraphica and the R language for processing. These tools are powerful and flexible, capable of handling large amounts of complex data and producing rich graphical and statistical results (18).

The citation and co-citation status of journals was analyzed with Citespace 6.3.R1, using vosviewer 1.6.19, we analyzed the co-occurrence of keywords, using ScimagoGraphica 1.0. 40, we analyzed the country, research institution, and author collaboration, and using R language, we analyzed the annual publication volume, country publication volume, and funding agency contribution, which in turn visually depicted the evolution path, distribution structure, and collaboration and research frontiers of TCM/atherosclerosis research. Co-occurrence network diagrams and keyword overlay diagrams were used to identify the main topics related to TCM/atherosclerosis. Further, we executed a descriptive scrutinization of the publications, inspecting the publication year, journal, country or region, institution, author, and citations to discern the contributions of active and productive individuals and teams.

## 3 Results

### 3.1 Annual publication trends in TCM/atherosclerosis

**Figure 2:**
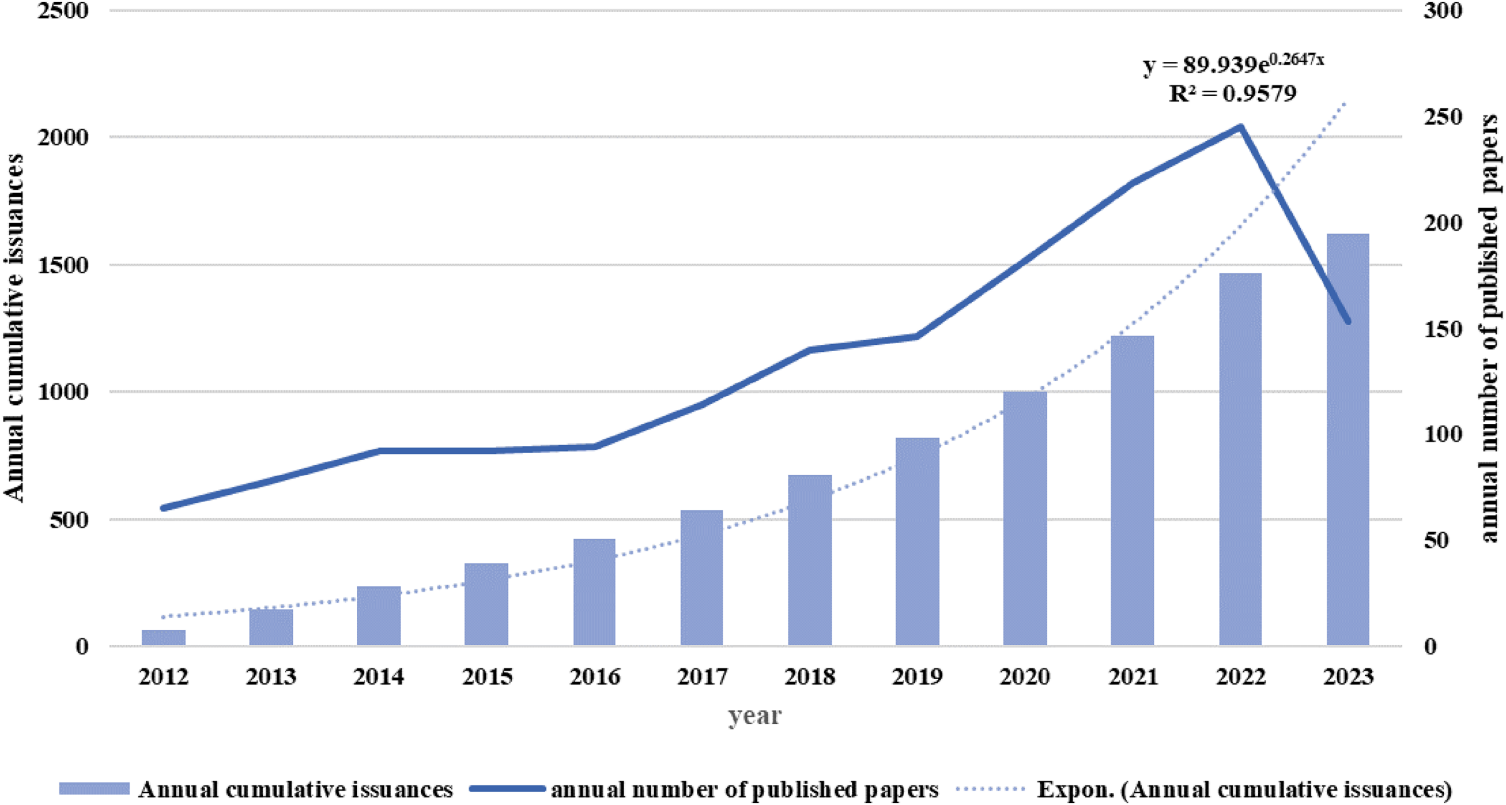
Annual number of published papers.

Present study encompasses an analysis of 1,623 scholarly articles pertaining to TCM/atherosclerosis. (As depicted in Figure 2), the volume of pertinent articles exhibited a gradual increase from 2012 to 2016, while witnessing a substantial surge from 2017 onwards, reaching its zenith in 2022. To anticipate continued growth in research publications, a curve-fitting model was constructed to predict future publication numbers.

### 3.2 Analysis of country/regions

**Figure 3:**
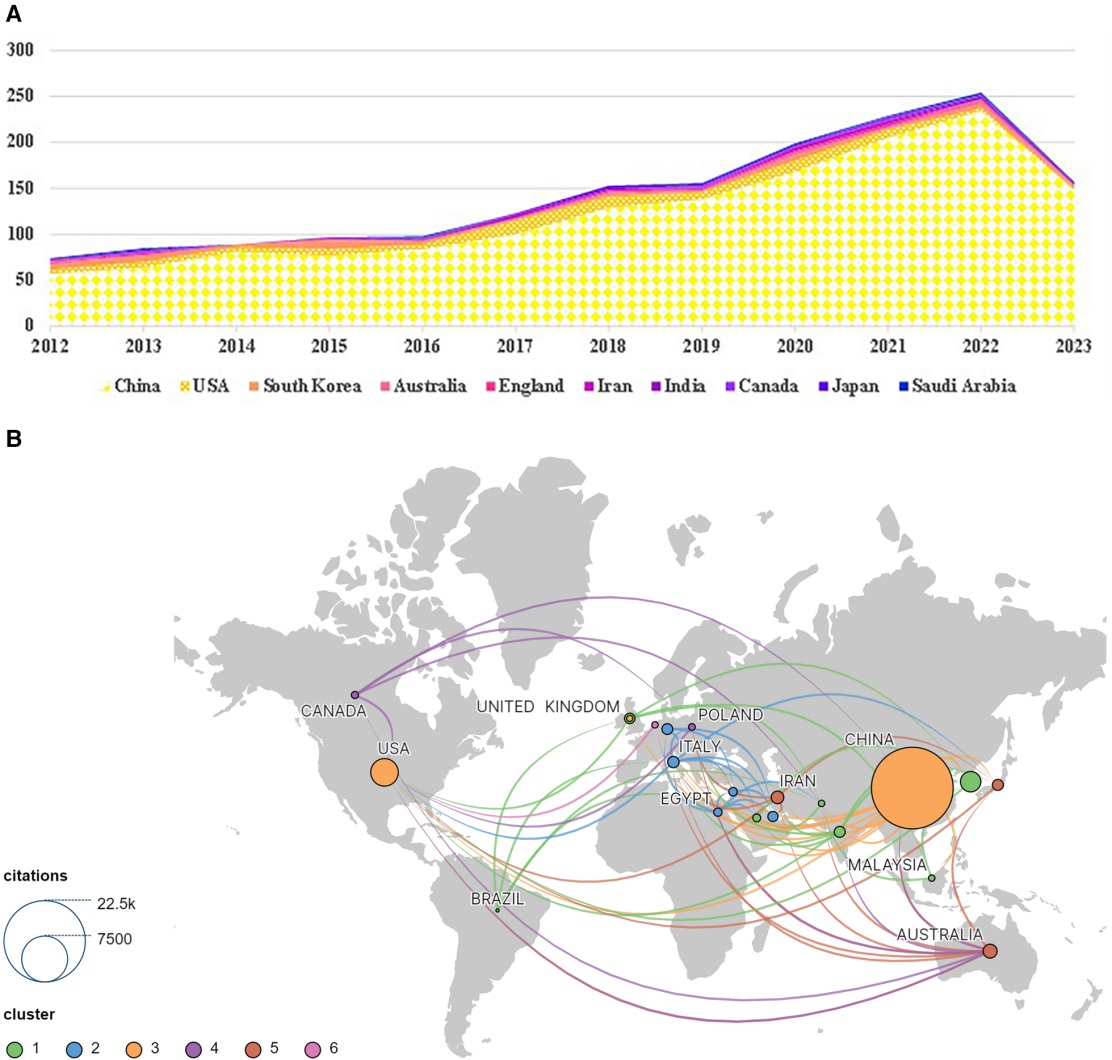
National/regional distribution and cooperation. (**A**) National annual publications map. (**B**) National scientific cooperation Map. Node size indicates the number of publications and colors indicate different clusters, Lines indicate partnerships.

A total of 61 countries/regions contributed to research on TCM and atherosclerosis. Table 1; Figure 3A lists the top 10 countries in terms of the number of publications. China held the primary position with 1,485 articles, constituting 91.497% of the total corpus. It was followed by the United States (88 articles), South Korea (50 articles), Australia (17 articles), the United Kingdom (15 articles), and Iran (14 articles). Despite the fact that Iran's global paper publication quantity is insubstantial, its articles possess an average citation rate of 36.36, superseding other countries. The research conducted in this field in Iran is widely recognized, as well as the depicted H-index value. The inter-nation collaboration graph 3(B) demonstrates that Asian countries maintain the highest level of cooperation and exchange, succeeded by North America, Australia, and China. China is the most active in cooperation and exchange in this field, followed by the United States, Australia, and Iran.

**Table 1:** Number of national publications.

Rank	Country	TP	TC	Average per item	H-index
1	China	1,485	21,545	14.51	58
2	USA	88	2,457	27.92	26
3	South Korea	50	1,345	26.9	19
4	Australia	17	629	37	7
5	England	15	362	24.13	10
6	Iran	14	509	36.36	10
7	India	13	399	30.69	9
8	Canada	11	166	15.09	6
9	Japan	11	396	36	7
10	Saudi Arabia	10	205	20.5	7

### 3.3 Analysis of funding agencies

**Figure 4:**
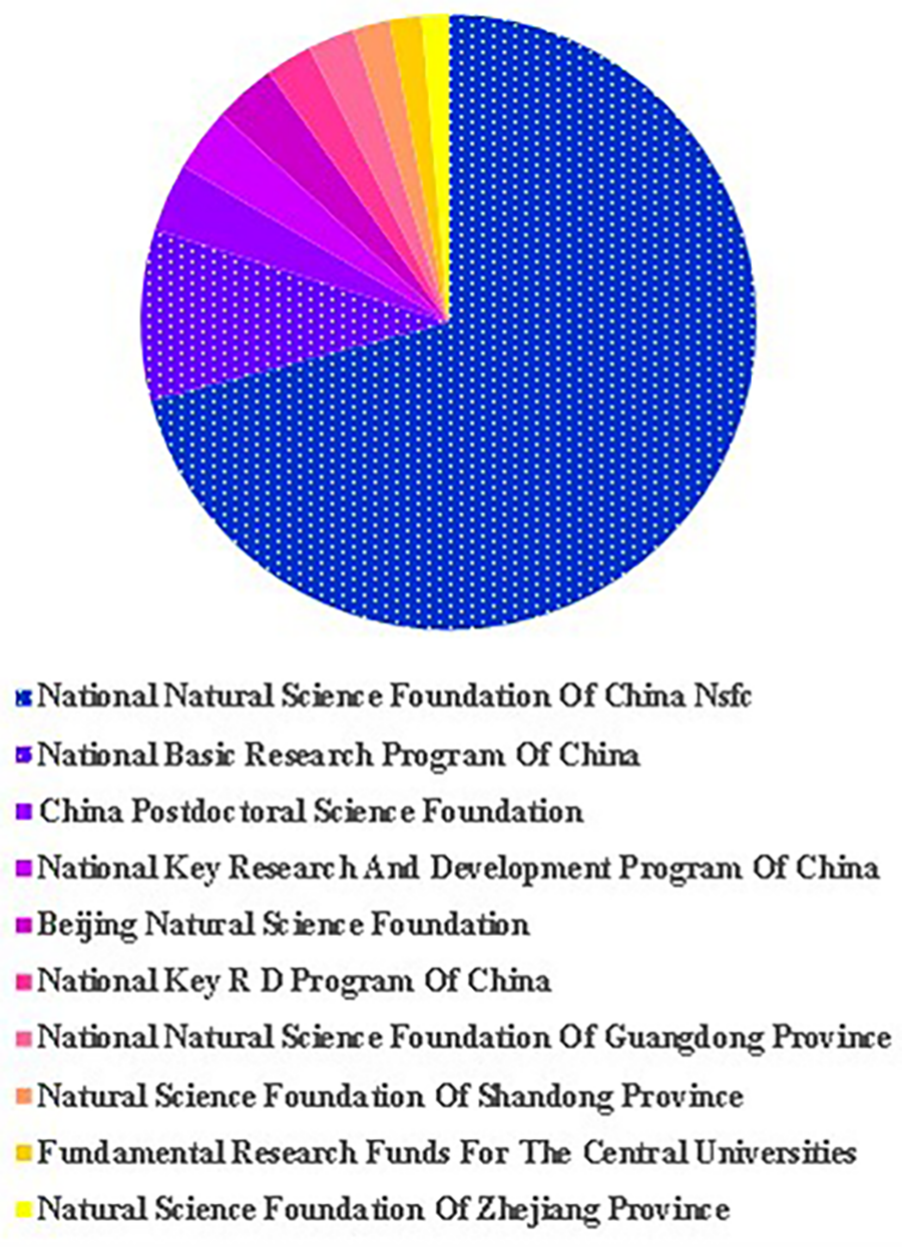
Major funding agencies.

Within the realm of TCM/atherosclerosis research, a diverse range of financing was endowed upon 221 investigative projects (Figure 4). China has distinguished itself by substantial investments in this domain, boasting one of the globally highest financing rates. Chinese entities consistently command the top ten slots relative to the quantity of financed projects. The investigation drew support from three principal funding bodies: the National Natural Science Foundation of China (NSFC) (*n* = 806), the National Basic Research Program of China (*n* = 102), and the China Postdoctoral Science Foundation (*n* = 41).

### 3.4 Analysis of institutions

Chinese research institutions have made significant contributions to this research area. Among investigative institutions, the CHINA ACADEMY OF CHINESE MEDICAL SCIENCES takes precedence (*n* = 219), succeeded by BEIJING UNIVERSITY OF CHINESE MEDICINE in second place (*n* = 217), and GUANGZHOU UNIVERSITY OF CHINESE MEDICINE securing the third position (*n* = 115) (Table 2).

**Table 2:** Number of publications by research institutions.

Rank	Research Institutions	PC	TC	Average per item	H-index
1	China Academy of Chinese Medical Sciences	219	2,717	12.41	26
2	Beijing University of Chinese Medicine	217	2,144	9.88	22
3	Guangzhou University of Chinese Medicine	115	1,089	9.47	17
4	Xiyuan Hospital	97	1,137	11.72	18
5	Shanghai University of Traditional Chinese Medicine	95	1,349	14.2	20
6	Tianjin University of Traditional Chinese Medicine	95	1,803	18.98	24
7	Guang'anmen Hospital	84	1,033	12.3	18
8	Capital Medical University	67	768	11.46	15
9	Nanjing University of Traditional Chinese Medicine	57	584	10.25	15
10	Chinese Academy of Medical Sciences Peking Union Medical College	56	897	16.02	18

**Figure 5:**
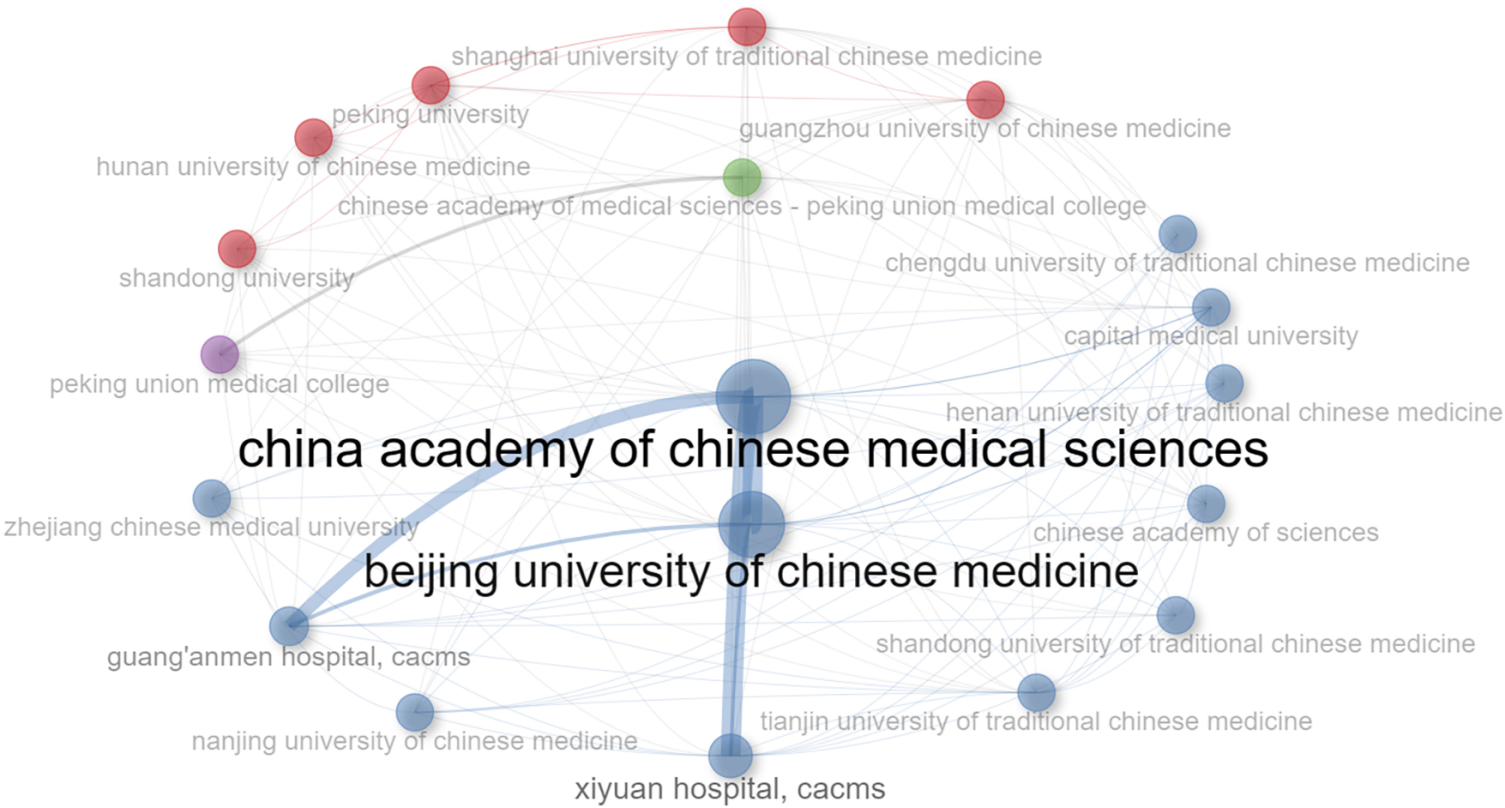
Distribution and collaboration trends. Coalition collaboration graph. Node size indicates the number of alliance publications. Different colors indicate clustering. Lines indicate collaboration between two affiliates, and thickness indicates frequency of collaboration; the coarser the collaboration, the higher the frequency.

Figure 5 delineates the dispersion and magnitude of the nodes, correspondingly representing the extent of contribution and the quantity of publications.The China Academy of Chinese Medical Sciences (CAMS) has the largest node and is located at the center of the circle, indicating the abundance of papers published by this institution in the field of TCM/atherosclerosis research and its central position in this research area. Beijing trails Shanghai closely, potentially suggesting the vibrancy of the northern region in this particular field of research.

In the cluster analysis of institutions, the blue conglomerate comprises the most substantial number of entities, signifying recurring mutual symbiotic relationships amongst them, with most being at the forefront of research institutions in China. The clusters colored in red represent institutions such as Hunan University, Peking University, Shanghai University, and Guangzhou University. This suggests that they maintain close cooperation networks in their respective research fields.

Concerning the visual depiction of the extent of collaboration, identifiable through line thickness, it's conspicuous that the China Academy of Chinese Medical Sciences cultivates frequent cooperation with Beijing, Guang'anmen, and Xiyuan hospitals, plausibly forming the bedrock for research in this area. In addition, the lines connecting Concordia Hospital of the Chinese Academy of Medical Sciences (CAMS) (purple) to Chinese Academy of Medical Sciences (CAMS), Guang'anmen, Beijing, and Capital University (CU) (green) are more prominent. This infers a heightened intensity of collaboration amongst these institutions.

### 3.5 Analysis of authors

**Figure 6:**
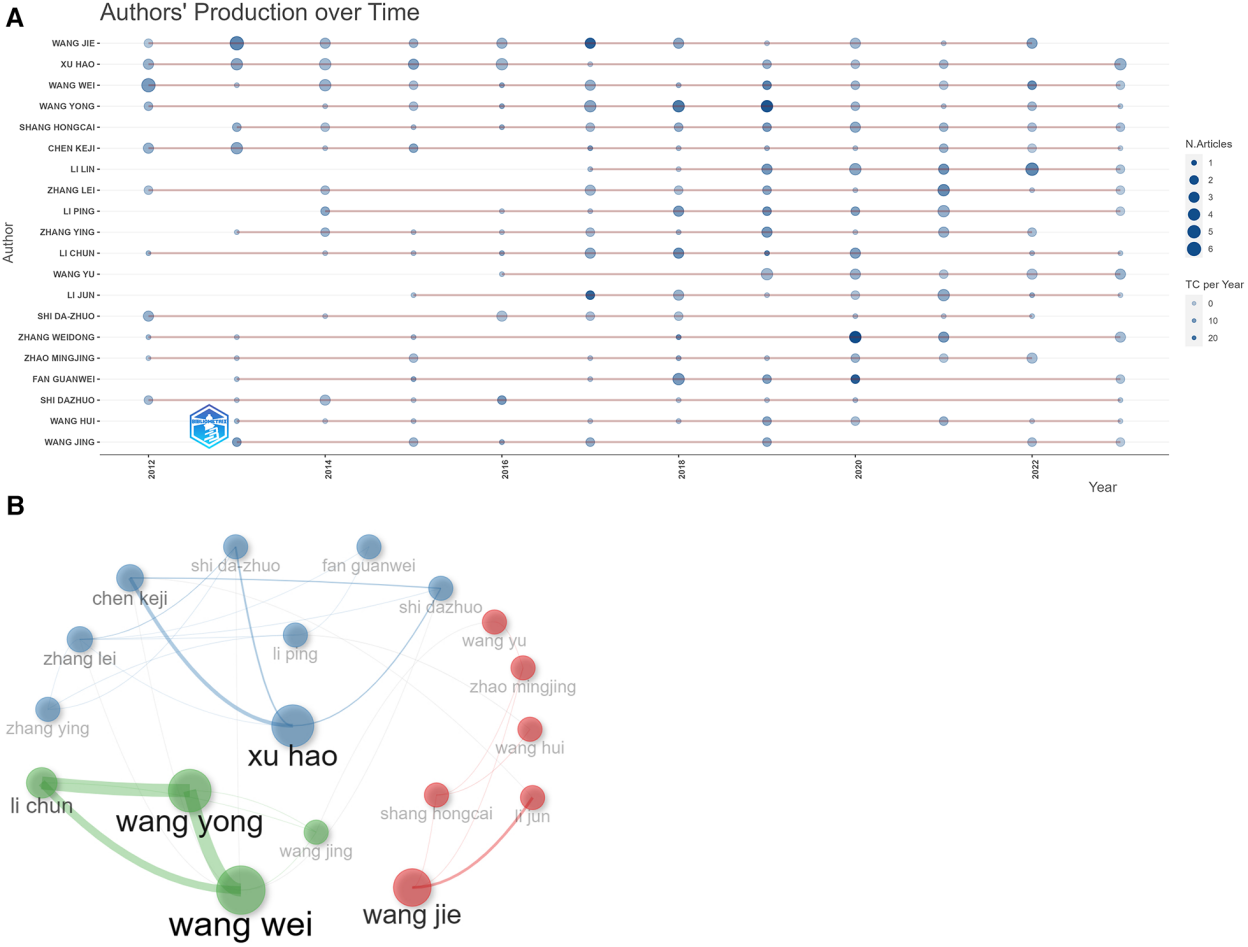
Author's writings and collaborations. (**A**) Graph of authors’ achievements over time. The size of the nodes indicates the number of articles published by the author, larger means more.The color indicates the number of citations, the darker the color, the more citations. (**B**) Author collaboration graph. The size of the nodes indicates the number of papers published by the authors. Different colors indicate clustering. Lines indicate the collaboration between two authors, and the thickness indicates the frequency of collaboration. The thicker the line, the higher the frequency of collaboration.

Pioneers from China have decidedly influenced this research discipline, with the prime ten scholars regarding global publications being unilaterally Chinese. As per Figure 6A, Shi Dazhuo (*n* = 27) leads the authorship, trailed by Chen Keji (*n* = 27) closely in the second position, and Xu Hao (*n* = 21) securing the third spot. Significantly, the research outcomes of Xu Suowen have achieved broad dissemination. Xu, Suowen has a high average citation frequency of 75.5 despite having only 14 publications, indicating excellent research quality and significant influence in the field. Figure 6B portrays the co-operative matrix among the topmost 18 authors in this research discipline. Xu, Hao is positioned at the center of the network, indicating his active role in promoting collaborative research. Wang Yong, Wang Wei, and Li Chun have engaged in extensive collaboration, culminating in the formation of a collaborative triad. Wang Yong and Wang Wei have worked most closely together, followed by Wang Yong and Li Chun. The graphic further delineates the formation of myriad author groupings. To guarantee the sustained progressive evolution of this discipline, fostering scholarly dialogue and alliances among these groupings becomes paramount. The data analysis presented here can help us understand which authors have had the broadest impact in this field. This may insinuate the potential instructive implications of their research orientation or methodology for fellow researchers.

### 3.6 Analysis of journals

The influence of each periodical and the pivotal focus of research in the discipline can be gauged by the volume of publications, mean citation rate, and H-index. Table 3 showcases the leading ten periodicals in this discipline, grounded on the quantity of published articles. JOURNAL OF ETHNOPHARMACOLOGY (*n* = 142) is ranked first, followed by FRONTIERS IN PHARMACOLOGY (*n* = 119) in second place, and CHINESE JOURNAL OF INTEGRATIVE MEDICINE (*n* = 67).

**Table 3:** Number of articles published in journals.

Rank	Journal Name	PC	TC	Average per item	H-index
1	Journal of Ethnopharmacology	142	3,138	22.1	32
2	Frontiers in Pharmacology	119	1,539	12.93	21
3	Chinese Journal of Integrative Medicine	67	480	7.16	13
4	Medicine	64	193	3.02	8
5	Biomedicine pharmacotherapy	61	947	15.52	19
6	Phytomedicine	58	797	13.74	17
7	Frontiers in Cardiovascular Medicine	29	164	5.66	6
8	BMC Complementary and Alternative Medicine	27	390	14.44	13
9	Molecular Medicine Reports	26	432	16.62	15
10	Oxidative Medicine and Cellular Longevity	22	357	16.23	13

The ramifications of the research were quantified utilizing the H-index (19). The journal with the highest number of articles published in the field of TCM/atherosclerosis was the “Journal of Ethnopharmacology”, which also had the highest average number of citations and H-index. This insinuates that the “Journal of Ethnopharmacology” exercises considerable vigor in this discipline, with its publications manifesting eminent quality and inference. Despite publishing a high number of articles, the journal Medicine has a relatively low average number of citations and H-index. This hints at the journal's possibly diminished standing within the academic cohort relative to other periodicals, despite its readiness to disseminate articles on TCM/atherosclerosis. Frontiers in Pharmacology, Biomedicine Pharmacotherapy, Phytomedicine, BMC Complementary and Alternative Medicine, Molecular Medicine Reports, and Oxidative Medicine and Cellular Longevity are notable for their average number of citations, which may suggest that their publications are of high quality and broad reach. Notwithstanding its circumscribed corpus of articles, the prominence of cardiovascular disease themes in ‘Frontiers in Cardiovascular Medicine’ lends it an invaluable status with field researchers.

### 3.7 Analysis of references

#### 3.7.1 Analysis of co-citation references

**Figure 7:**
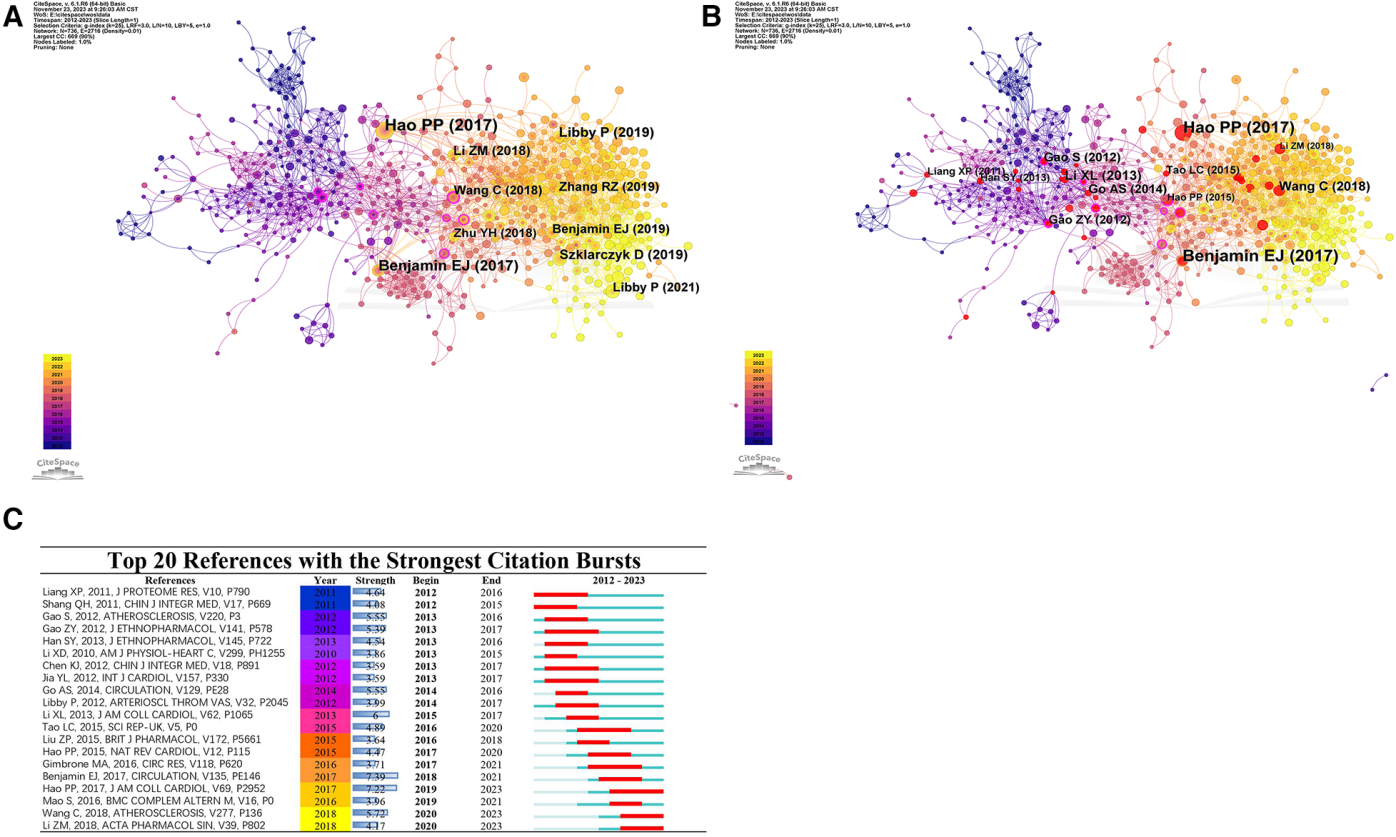
Reference analysis. (**A**) Top 10 charts of total cited literature. Node size indicates frequency and color indicates different years. (**B**) Top 10 bursts of co-cited literature. Red nodes indicate bursts of literature. (**C**) Graph of the top 20 bursts of co-cited literature. In the 2012-2023 column, red indicates the year of the outbreak, and green indicates the year when the literature started to be cited until it declined.

Co-citation references serve as a robust metric for estimating the correlation between scholarly works. Two or more papers are said to have a co-citation relationship when they are cited together by one or more subsequent papers (20). The most notable feature of CiteSpace is its ability to identify co-citation references (21). In the present investigation, we employed CiteSpace 6.2.R5 with the scaling factor k set at 20, consequently identifying 42 substantial and impactful citations. The g index facilitated the identification of the ten most crucial papers within the co-citation network of this discipline (Figure 7A). These papers have a high citation frequency and are closely related to TCM research on atherosclerosis.

Using Hao PP as an example, the study focuses on evaluating and analyzing the efficacy and safety of traditional Chinese medicine (TCM) in treating cardiovascular diseases. Furthermore, the investigation delves into probing the pharmacological implications and potential mechanistic contributions of active constituents in TCM on the cardiovascular system. The findings infer the potential utilisation of TCM as a supplemental and alternative strategy in primary and secondary prophylaxis against cardiovascular ailments (22). Benjamin EJ assimilates and underscores the most recent epidemiological data on heart disease, stroke, and cardiovascular risk determinates authorised by the American Heart Association (23). Libby P's study comprehensively describes the occurrence, development, and complications of atherosclerosis, as well as its preventive and therapeutic measures, providing valuable theoretical support and insights for future research in the field of TCM/atherosclerosis (24). These studies have provided valuable insights and analysis on the use of Traditional Chinese Medicine (TCM) in treating cardiovascular diseases, particularly atherosclerosis. They manifest the curative prospects of TCM and furnish significant insights for prophylaxis and management of cardiovascular conditions.

#### 3.7.2 Analysis of burst references

A reference burst pertains to the episodic surge in citation frequency a particular paper undergoes over a given temporal span. Analyzing these papers can help identify research trends during a certain time period and predict future research directions. Figure 7B presents the 20 references manifesting the most profound citation bursts within the sphere of Chinese Medicine/Atherosclerosis, which encompasses 8 articles classified under reviews.

Figure 7C illustrates that citation bursts conventionally endure for an interval of 3–5 years, presenting outliers such as “(25)” and “(15)”, with their citation surges projecting till 2023. The publications exhibiting the greatest citation potency are “(26)”, “(25)”, and “(15)”, possessing citation intensities of 7.39, 7.22, and 5.72, in that order. These robust citation magnitudes could denote substantial research value and pervasive influence of these papers within their affiliated research domains. This scholarly investigation centres on analysing the theoretical mechanisms implicated in the application of Traditional Chinese Medicine (TCM) for the treatment of atherosclerosis. Accommodating dyslipidemia, agents such as Lipid-lowering and Tongluo capsule, Danshen Geran capsule, and Stop Lipotex capsule demonstrate efficacious lipid-curbing activities and pose as tenable substitutes for Western pharmaceuticals (22). In a methodical discourse, Li ZM pontificates on the cardiovascular implications and therapeutic viability of the chief pharmacologically active elements in Danshen (27).

Wang C put forth a thorough encapsulation of atherosclerosis pathophysiology as perceived in both Chinese and Western medicinal practices. Moreover, the efficacy of Chinese herbal medicine in addressing atherosclerosis was appraised, accentuating its merits and limitations (15). This scholarly endeavour furnishes consequential perspectives instrumental for the advancement of Chinese medicine. The citation surge observed in certain articles from the 2012 era encompasses a study demonstrating the efficacy of the Chinese medicine Shuanglong formula in treating myocardial infarction in rats (28), as well as an investigation assessing the safety and effectiveness of Xiongshao capsule in preventing restenosis in elderly patients with coronary artery disease following percutaneous coronary intervention (29). This may indicate that these early studies played a significant role in atherosclerosis research within traditional Chinese medicine and exerted a profound impact on subsequent studies. This amassed tableau epitomizes notable strides and critical milestones in the realm that are poised to navigate and contour prospective research trajectories.

#### 3.7.3 Analysis of high LCS score papers

Table 4 showcases the ten most extensively cited works within the landscape of Chinese medicine and atherosclerosis research, as referenced by local academicians. The resourced articles traverse thematic areas encompassing Chinese medicine research, intervention modalities for atherosclerotic diseases, pathology, and network-oriented pharmacological studies.

**Table 4:** Top 10 locally cited references.

Cited articles	Number of citations
Ru et al. (30)	72
Hao et al. (22)	67
Ross (31)	59
Li et al. (32)	46
Shannon et al. (33)	44
Benjamin et al. (26)	42
Li and Zhang (34)	42
Zhou et al. (35)	38
Gao et al. (36)	36
Hopkins (37)	35

The article “TCMSP: A Database of Systems Pharmacology for Drug Discovery from Herbal Medicines” received the highest number of citations from local scholars (72 times) (30), followed by “Traditional Chinese Medicine for Cardiovascular Disease: Evidence and Potential Mechanisms,” and “Atherosclerosis: An Inflammatory Disease,” with 67 and 59 citations respectively (22, 31).

Ru J discussed the importance of establishing a systems pharmacology platform (TCMSP) to gain a deeper understanding of the mechanisms of action of herbal medicines and drive the discovery of new drugs. He underscored the import of rigorous clinical trial designs for the precise assessment of the prolonged effectiveness of TCM in patients implicated with cardiovascular pathologies (30). Certain herbs, such as danshen and qili qiangxin capsules, have demonstrated positive clinical effects. Danshen is commonly used in the treatment of cardiovascular diseases (35), and its active ingredient, tanshinone IIA, has been shown to have preventive effects against atherosclerosis and other cardiovascular diseases (36). Supplementary to this, qili qiangxin capsules have elicited beneficial impacts on patients enduring chronic cardiac failure (32).

Shannon P employed comprehensive investigations of herbal components at a cellular level, network examinations of biomolecular interplays via software utilities like Cytoscape, and amalgamated biomolecular interconnection networks with large-scale expression data and various molecular states, all to unveil the operative principles of herbs (33).

Benjamin EJ probed into the domain of heart disease and stroke with a statistical lens (26, 38). Hao P evaluated the efficacy and safety of Chinese medicine in treating cardiovascular diseases, as well as the pharmacological effects and potential mechanisms of active herbal ingredients on the cardiovascular system (22). Ross R scrutinized the development trajectory of atherosclerosis from the standpoint of inflammatory responses (31).

Cyberpharmacology, as a nascent discipline, leans on vast data resources and computational methodologies to dissect the intricate data networks between pharmaceutical compounds and their targets, pathways, genes, proteins, and other molecular entities in the organism. It further investigates the implications of these interactions on medicinal effectiveness, toxicity potential, and metabolic behavior. Hopkins AL used cyberpharmacology to study the clinical efficacy of drugs (39, 40). This approach focuses on the multi-target effects of drugs, with the aim of enhancing their efficacy and minimizing toxicity (37). Li S's study suggests that the TCM network pharmacology approach can provide a new research framework for the transition from empirical to evidence-based medicine in TCM (34).

These prominently recognized academic papers in the realm of TCM and atherosclerosis have exerted substantial influence and are envisaged to propel the course of renascent research undertakings as well as the governance of pertinent health conditions.

#### 3.7.4 Analysis of cluster dependencies of cited references

Cluster analysis operates as a statistical strategy that systematizes items bearing marked similarity into distinct groupings or clusters. Its principal objective is to maximize the homogeneity amongst elements within the same cluster while ensuring a marked divergence between objects belonging to separate clusters (41).

The catalogue of 1,623 articles underwent analytical scrutinization via CiteSpace reference clustering. The nomenclature attributed to each cluster, courtesy of several algorithms (LSI, LLR, and MI), reflects the encapsulated subject matter within the clustered topics.

Cluster 0 bears the thematic congregation identified as ‘Therapeutic Potential and Research Advances in Traditional Chinese Medicine and Cyberpharmacology.’ Concentrated within this group is a collection of articles devoting attention to the potential operative pathways of traditional Chinese medicine in managing coronary artery disease, spanning the SREBP1 pathway, the manipulation of intestinal flora, and the moderation of lipid metabolic processes.

Cluster 1 focuses on the treatment of myocardial ischemia/reperfusion injury and heart failure with Chinese medicines, specifically “Yongan Tang” and “Danshen Tang”.

Cluster 2 focuses on the research of Chinese medicine treatment of coronary heart disease, with special attention to the mechanism of action of the five prescriptions of coronary heart disease and network pharmacology, as well as research on ginseng-mai injection.

Cluster 3 focuses on cardiac function, cardiac remodeling, and murine models of heart failure, and explores the treatment of cardiovascular diseases with Chinese medicine.

Cluster 4 focuses on evaluating the efficacy of Chinese herbs and Danhong injection in treating cardiovascular diseases, highlighting various herbal combination therapies.

Cluster 5 examines the treatment of blood stasis, including advanced applications such as blood-activating herbs and platelet proteomics, as well as the efficacy of unstable angina and compound danshen.

The primary theme of Cluster 6 materialized through clinical investigations on myocardial infarction, ventricular arrhythmias, and coronary heart disease. This category additionally enveloped studies on gold-red herbs and Chinese therapeutic protocols for addressing cardiovascular diseases.

The outcomes from the cluster analysis of this corpus of literature underscore the criticality of herbal medicines in addressing cardiovascular ailments. Moreover, the growing prominence of network pharmacology in the realm of herbal medicine studies manifests considerable potential for ensuing research and clinical applications.

### 3.8 Analysis of keywords in the TCM/atherosclerosis

#### 3.8.1 Frequencies keywords and bursts keywords

Utilizing VOS software, a keyword contribution network diagram orientated towards TCM/atherosclerosis papers was engineered. It was determined that a minimal keyword occurrence of six instances was necessary to preserve readability and aesthetic presentation. A total of 4,370 keywords were extracted from the imported papers, and the most frequently used keywords were: This text discusses the relationship between atherosclerosis, oxidative stress, inflammation, activation, myocardial infarction, disease, apoptosis, mechanisms, injury, traditional Chinese medicine, inhibition, dysfunction, and NF-kappa-B in the context of heart disease and cardiovascular disease. Furthermore, it delves into the role of ischemia-reperfusion damage and the prospective avenues for safeguarding against such harm. These high-frequency keywords highlight the top priority of research in the field of traditional Chinese medicine (TCM) and atherosclerosis. They cover a wide range of areas, including the causes and processes of atherosclerosis, cardiovascular-related physiological and pathological changes, as well as the mechanisms, pathways, and protein expression related to TCM treatment of atherosclerosis.

**Figure 8:**
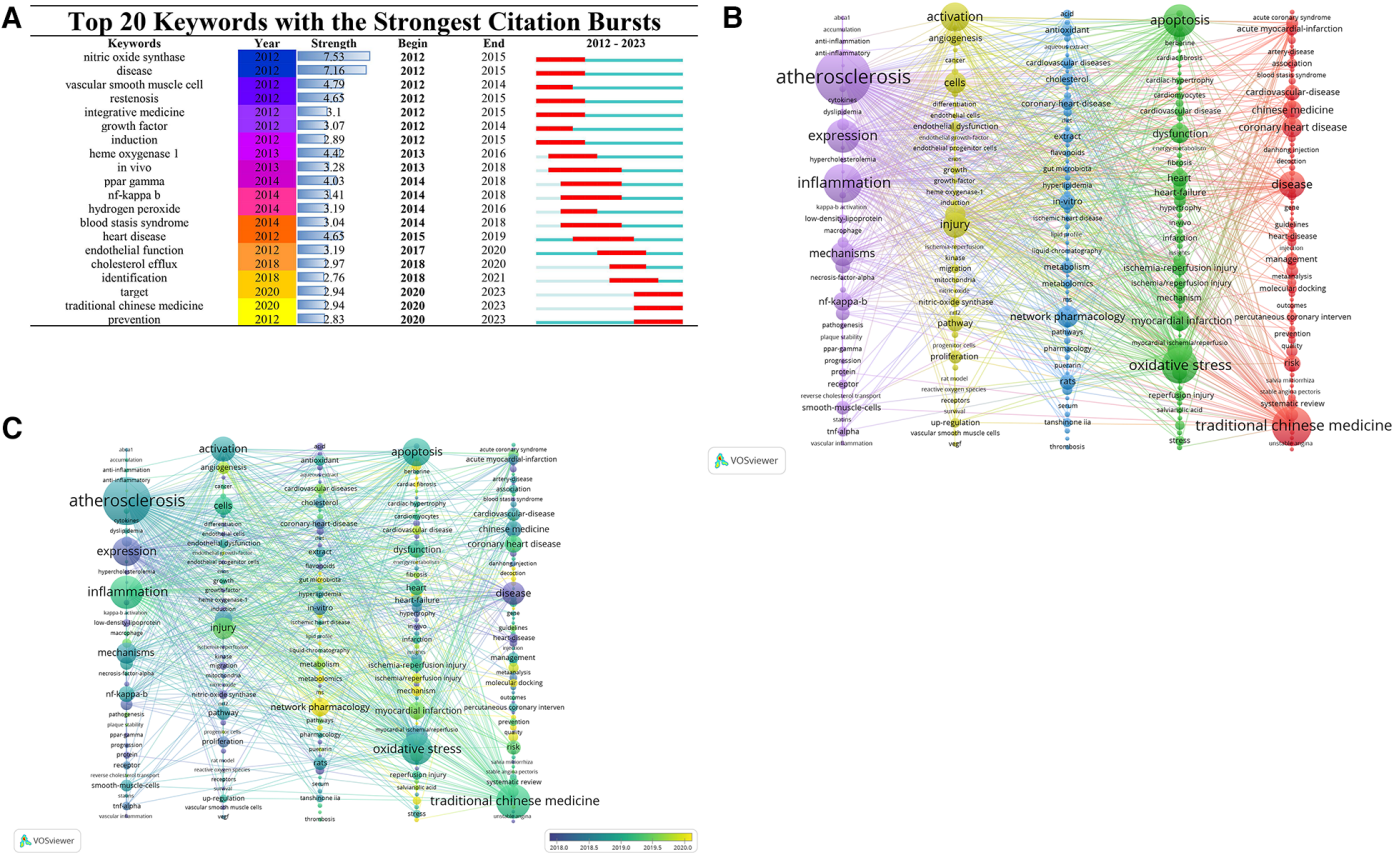
Keyword trends. (**A**) Keyword burst graph. (**B**) Keyword co-occurrence graph. Node size indicates the frequency of keyword occurrence; the larger the node, the higher the frequency of occurrence. Node colors indicate different clusters. Lines indicate co-occurrence of two keywords in the same paper or papers. (**C**) Keyword time overlay graph. Different colors of the nodes indicate different years; the rest is consistent with (**B**).

Such keywords possess the capacity to elucidate the particular concerns of the scientific community, proving instrumental in investigating research frontiers, forecasting trajectories, and uncovering research hotspots. The Figure 8A contains the compilation of the discerned keywords. This study flagged 20 “outbreak” keywords, essentially terms witnessing a substantial upswing in usage within delineated temporal brackets.

Each of the 20 keywords featured in this diagram registers an outbreak intensity of at least 2.76, hitting a maximum intensity at 7.53.The crux of decoding broad research patterns lies in keyword analysis. These earmarked words encapsulate the salience of atherosclerosis within scientific inquiries and embody the diversity of research advancement it has engendered. The keywords can be analyzed for each period, starting with

2012–2015: During this period, there has been a high level of interest in the keywords “nitric oxide synthase” and “disease”, indicating a focus on the role of nitric oxide synthase in the pathology of atherosclerosis. Nitric oxide synthase is an enzyme that synthesizes nitric oxide in endothelial cells and is important for regulating vascular tone and inhibiting platelet aggregation. Nitric oxide (NO) is a soluble gas that is synthesized by calcium-calmodulin-dependent NOS enzymes catalyzed by NOS from the amino acid L-arginine in endothelial cells (42). It regulates vasodilation, modulates local cell growth, and protects blood vessels from damage caused by blood platelets and cellular circulation. Therefore, it plays a key role in normal vascular endothelial function, lowers blood pressure, and promotes reperfusion and cytoprotection (43). Research has demonstrated that the Glu298Asp polymorphism (rs1799983) found in the endothelial nitric oxide synthase (NOS3) gene is linked to reduced levels of circulating nitric oxide and an elevated risk of coronary heart disease (44). The incorporation of terms such as “vascular smooth muscle cell”, “restenosis”, and “integrative medicine” underscore crucial considerations in shaping therapeutic frameworks. These encompass aspects like curbing the disproportionate proliferation of vascular smooth muscle cells, mitigating restenosis, and advocating for integrative medicine.

During the five-year span from 2013 to 2018, a surge of interest amongst researches was noted in unveiling novel therapeutic targets and delving deeper into the pathophysiological intricacies of atherosclerosis via the examination of “ppar gamma”, “nf-kappa b”, and “hydrogen peroxide”. The appearance of the keywords “heme oxygenase 1” and “blood stasis syndrome” may suggest the application of Traditional Chinese Medicine (TCM) theories in this field, such as treating atherosclerosis by improving blood stasis syndrome.

Between 2015 and 2019, the phrase “heart disease” began commanding broader attention, being acknowledged as a significant trigger of atherosclerosis. Concurrently, the maintenance of endothelial function started gaining increasing acknowledgment, substantiated by the emergence of the phrase “endothelial function”.

Spanning from 2018 to 2021, an upsurge was spotted in the intensity of the keyword “cholesterol efflux”, implying a prioritization by investigators towards the regulation of lipid metabolism to curb and manage atherosclerosis. The appearance of the keyword ‘identification’ may indicate a growing recognition of the importance of individualized treatment through fine-tuned diagnosis.

Progressing to the period of 2020–2023, we noticed a pivot in the research thrust towards drug targeting, leveraging traditional Chinese medicine, and proactively circumventing atherosclerosis. The rise of the keywords “target”, “traditional Chinese medicine”, and “prevention” suggests this shift.

Collectively, these particular outbreak keywords along with their intensity are reflective of the research concentration encompassing diverse facets of atherosclerosis, from its etiology and pathophysiological mechanisms to diagnosis, circumvention, and therapy. The research on the treatment of atherosclerosis with TCM has evolved from early investigations on etiology and pathophysiological mechanisms to later emphasis on diagnosis and prevention, as well as the role of TCM in treatment. This indicates a trend towards individualization and diversification.

#### 3.8.2 Analysis of keywords co-occurrence in the TCM/atherosclerosis

The keyword co-occurrence network diagram, a well-utilized bibliometric instrument, visually encapsulates the progress, thematic exploration, and intertwined relationships within one or several fields of research. Its intuitive and precise presentation allows for the statistical analysis of keywords in a large number of documents, revealing unbiased and objective hotspots and trends in research. Figure 8B exhibits results of the cluster analysis, allocating individual keywords into five clusters grounded on their recurrence amplitude in their respective domains.

The first category, #1 (purple theme), mainly covers topics related to heart disease, such as acute heart disease, coronary artery disease, myocardial infarction, and so on. Additionally, featuring terms like “Chinese drugs” and “percutaneous coronary intervention”, it is inferred that studies within this theme could be centered on investigating the efficacy of Chinese medicaments in cardiovascular disease management.

Category #2 (Yellow Theme) zeroes in on fundamental investigations of cardiovascular conditions, encapsulating aspects such as cardiomyocyte apoptosis, myocardial hypertrophy, myocardial fibrosis, among others. The keywords in this section are related to the pathophysiological mechanisms of heart diseases and may indicate in-depth studies on the mechanisms of development and progression of these diseases.

Category #3 (Blue Theme) is centered on notions including antioxidants, lipid metabolism, biomarkers, and so forth. These terms bear significance in evaluating drug effectivity as well as in assessing disease diagnosis and prognosis.

Category #4 (Green Theme), the fourth segment, lays emphasis on elucidating fundamental concepts and intricate mechanisms within biochemistry and molecular biology, taking into account elements like endothelial cell malfunction, inflammation response, and nuclear transcription factor kappa B activation.

Conversely, category #5 (Red Theme), the fifth cluster, predominantly delves into rudimentary cell biology, encompassing notions like pathway management, genetic composition, inflammatory reactions, and corresponding signal conduits. Specifics may relate to cellular regulation, molecular biology, biochemical mechanisms, etc.

The application of time series in assessing keyword co-occurrence network diagrams adeptly highlights the progressive trajectory of research foci and their emerging patterns. This bears significant implications for strategizing and decision-making in prospective research endeavors. The VOSviewer tool was harnessed to render distinct research domains visible, relying on a color-coding schema premised on the average publication year in structuring the diagram 8(C). Historically, the research lens was trained on concepts encompassing expression, disease manifestation, atherosclerosis, activation, apoptosis, oxidative stress, classical Chinese medicine applications, and injury. However, the current research concentrates on network pharmacology, mechanism, quality, and gut microbiota as the key areas of study.

In sum, the exploration of atherosclerosis within the ambit of TCM has adeptly amalgamated elements of molecular biology and informatics. These new approaches offer fresh insights into the disease's systems biology and form a foundation for modernizing TCM.

## 4 Discussion

### 4.1 Growing trends in TCM/atherosclerosis literature

Figure 2 illustrates an ascendant trend in the annually published literature volume, progressing from 65 in 2012 to 245 in 2022, reflecting a burgeoning interest in TCM/atherosclerosis investigation within the past ten years. Research in this area has experienced significant growth since 2016. The number of publications increased from 94 in 2016 to 245 in 2022. A plausible explanation for this expansion could be the amplified research funding contributions by China and the United States, as delineated in Figure 3A. This growth trend may indicate increased attention to the disease in society in recent years and may also reflect significant advances in research tools and techniques in this field.

Outbreak Figure 8A conveys the message that atherosclerosis, the predominant etiology of heart disease, has drawn profound scholarly engagement. Between 2014 and 2018, research on blood stasis syndrome gained popularity, particularly in 2015 and 2016. The vast majority of investigations featuring this syndrome were spearheaded in China, with academics there deeming it an influential variant of the coronary heart disease syndromes (45). Chinese academics have intensified their endeavors to manage blood stasis syndrome utilizing traditional Chinese medicine, underlined by a contemporaneous update in diagnostic standards for blood stasis syndrome (46). Metabolomic examinations have unearthed that perturbations in glucose and lipid metabolic processes primary instigate blood stasis in the context of coronary heart disease (47). A multitude of Chinese medicinal treatments has been assessed for their efficacy in treating angina pectoris by promoting hemodynamic circulation and ameliorating blood stasis. This evaluation serves as the basis for more rigorous clinical trials (48).

Analyzing the co-cited literature graph 7(B), the most influential studies are those by Benjamin EJ and Hao PP in 2017, and Wang C in 2018. These articles primarily shed light on the interplay between TCM and cardiovascular ailments, and they wield substantial scholarly value and influence. Using Benjamin EJ as an example, the statistics on heart disease and stroke were updated for several years (23, 26, 49, 50). Hao PP assessed the efficacy and adverse effects of traditional Chinese medicine (TCM) in treating cardiovascular diseases by reviewing 68 randomized controlled trials involving 16,171 patients (25). Wang C pointed out the dilemma of treating atherosclerosis with TCM at that time (15). These papers have great value and influence. These publications have substantially propelled the proliferation and knowledge dissemination pertinent to Traditional Chinese Medicine (TCM).

Spanning the years 2019–2022, there was a marked ascension in the annual literary production, particularly evident in China, escalating from 138 pieces in 2019 to 234 in 2022. This observable trend could be associated with the growing emphasis on the role of the endothelium and the metabolic pathways implicated in cholesterol regulation. Figure 8A depicts the keyword outbreak. Endothelial cells are crucial in the development of atherosclerosis. In a high-impact article published in 2019, the properties of endothelial cells were reviewed as central sensors of friction generated by blood flow (51). Maintaining cholesterol homeostasis is essential for the proper functioning of the cardiovascular system (52), and managing lipids can prevent atherosclerotic cardiovascular disease (53). The advent of the coronavirus pandemic precipitated a surge in field-related research, owing largely to the solid correlation unearthed by investigators between coronavirus-linked disorders and cardiovascular diseases (54).

The annual publication volume for the year 2023 shows a slight decline. This may be due to the fact that the year has not yet ended and many research papers are still being prepared for publication. An alternate explanation could be that the research paradigm shift has been impeded by technical challenges and finds itself wanting in terms of cutting-edge theoretical and technological backing. Nonetheless, adopting a macroscopic viewpoint, the volume of academic publications within this research sphere has swelled considerably from 65 in 2012 to a hefty 1,623 in 2024, signifying a brisk developmental velocity and substantial academically valued outcomes in the TCM/atherosclerosis field. It is expected that research in this field will continue to grow in the coming years.

### 4.2 Status and characteristics of the TCM/atherosclerosis literature

Within the domain of TCM and atherosclerosis investigations, China has made a markedly superior contribution in terms of research publications than any other nation, boasting the most significant H index score of 58. This demonstrates China's active and dominant research position in this field. Of particular note, the corpus of China's scholarly outputs has demonstrated a steadfast ascending pattern over the period spanning 2012–2023. The United States and South Korea secured the second and third positions correspondingly, albeit their cumulative publication tally noticeably lags behind China's. Concurrently, nations such as Australia, the United Kingdom, Iran, and Japan manifest relatively meagre publication volumes in this sphere of study. Upon examining the average number of citations per item, it is evident that countries with low publication volumes, such as Australia, Iran, and Japan, have surprisingly high average citation numbers. This suggests that the quality and impact of papers in the field of Chinese medicine and atherosclerosis research in these countries are comparatively high.

The National Natural Science Foundation of China (NNSFC) has been a notable backbone for Chinese investigations within the realm of traditional Chinese medicine (TCM) for atherosclerosis, underpinning its irreplaceable role in both foundational and applied research initiatives. The foundation's establishment by the Chinese central government demonstrates China's deep interest and importance in this research field.

Moreover, the conspicuous involvement of national funds, exemplified by programs such as the “National Basic Research Program of China” and the “National Key Research and Development Program of China”, underscores China's resilient endorsement of foundational research into atherosclerosis management via Chinese medicine. This illustrates that when theoretical breakthroughs and practical exploration are combined, the national key research and development program will respond in a more open and comprehensive manner, allowing for the treatment of atherosclerosis in future clinical practice. This demonstrates China's support for basic research on atherosclerosis treatment. The National Key Research and Development Program of China responds with an open and comprehensive attitude when frequent theoretical breakthroughs are intertwined with practical explorations. This methodology aspires to catapult significant advancements in clinical practice for future atherosclerosis management. Other local-level funds, such as Beijing and Shandong, are also actively involved in supporting research in this field. In summation, this manuscript underscores the broad-ranging support and spirited involvement at national, provincial, and academic tiers in the terrain of TCM-aided atherosclerosis treatment research. It proves that this field has important scientific and social value.

In terms of publication volume, the China Academy of Chinese Medical Sciences (CAMS), Beijing University of Chinese Medicine (BUCM), and Guangzhou University of Chinese Medicine (GZUCM) are the most active institutions in the field of Chinese medicine and atherosclerosis, with the highest number of publications ranking in the top three places. The China Academy of Chinese Medical Sciences represents a vanguard in this research domain, cultivating wide-ranging collaborations with other research institutions, notably Beijing University of Chinese Medicine, Guang Anmen Hospital CACMS, and Xiyuan Hospital CACMS. Nonetheless, these collaborative pursuits are predominantly confined to a domestic purview, thus failing to engage in substantive discourse and collaboration with international scholars.

The investigation unveiled that scholarly periodicals, namely “Journal of Ethnopharmacology”, “Frontiers in Pharmacology”, and “Chinese Journal of Integrative Medicine”, claim the highest number of TCM/atherosclerosis research publications. These journals have made significant contributions to the field, and researchers prefer to publish their findings in them.

China is at the frontier of TCM/atherosclerosis research, underscored by substantial financial backing and a plethora of publications from esteemed research institutions and journals. It is expected that research momentum in this area will continue to grow, and more major breakthroughs are expected to be realized.

The top ten authors in this field are all from China, with Shi Dazhuo and Chen Keji leading the way with 27 publications. Topping the ranks with 27 scholarly pieces are Shi Dazhuo and Chen Keji, trailed closely by Xu Hao with a tally of 26 publications. Xu Hao has been the most active in seeking collaborations with others. Xu Suowen's research has the highest number of citations and mainly focuses on the effects of active monomers of traditional Chinese medicines on atherosclerosis. For instance, Danshinone (Tanshinone II-A) can combat atherosclerosis by inhibiting the NF-κB signaling pathway and reducing phagocytosis of oxidized LDL by macrophages, thereby inhibiting the formation of foam cells (55).

Furthermore, botanicals and their bioactive derivatives, which include salvinorin A and salvinorin B, exhibit efficacy in cholesterol regulation, thereby mitigating atherosclerosis risks (56). Plant and floral components harbor flavonoids (e.g., flavanones), polyphenols (e.g., peony dermatophyllin), and quinones (e.g., ophiopogonin), which serve dual roles in quelling inflammatory responses while enhancing endothelial cell functionality, discouraging platelet accumulation, and exerting notable anti-atherosclerotic impacts (57–59).

As elucidated by Guanwei Fan's research, danshen, and its potent constituent, tanshinone II-A, hold prominent therapeutic utility in managing cardiovascular ailments. Tanshin can enhance vasodilatory performance and forestall endothelial dysfunction, thereby affording substantial cardiovascular safeguarding (60). Additionally, tanshinone II-A promotes vasodilation by activating ion channels in cardiovascular smooth muscle cells (61). For instance, Ganoderma lucidum triterpenes, a sole active component in the herb, prevent atherosclerosis and vascular lining by regulating cholesterol metabolism and exhibiting anti-inflammatory effects (62). Compound concoctions, encompassing Deep Vein Formula, Qidan Lixin Wan, and Ganoderma Lucidum Spore Powder, likewise attest efficacy in ameliorating cardiovascular maladies. These formulas improve lipid metabolism, reduce inflammation, promote vascular remodeling, protect the myocardium, and even prevent atherosclerosis and liming (63, 64).

Whether deployed singly or synergistically, herbal remedies and combined pharmaceuticals have showcased substantial benefits in both mitigating and treating cardiovascular diseases. Their therapeutic effects are mainly realized through the regulation of lipid metabolism, anti-inflammation, improvement of endothelial function, and regulation of cholesterol metabolism.

### 4.3 Research hotspots and development analysis

**Figure 9:**
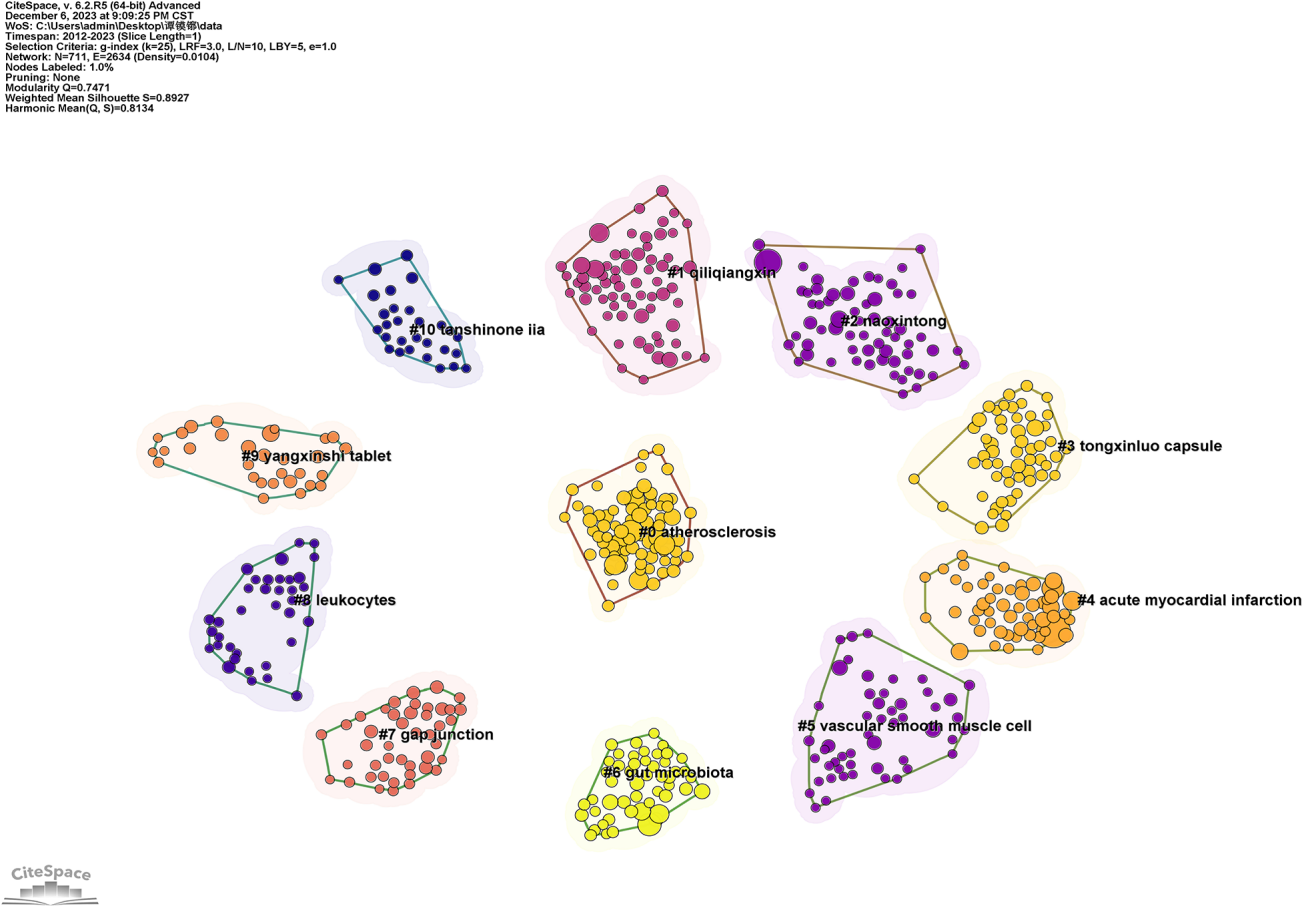
Co-referenced network graph.

The frequent recurrence of specific keywords proves instrumental in discerning key themes within a field, in tandem with cluster analysis that unpacks the spread of primary research substance pertinent to the topic. Figure 9 illustrates the findings of high-frequency keyword co-occurrence and cluster analysis, unraveling crucial concepts and principal constituents of the overlap between TCM and atherosclerosis. This encompasses multiple dimensions, such as disease characterization and pathogenic mechanisms, theoretical constructs of TCM, and its specific evidence-supported treatments, potential of herbal interventions, and manipulation of gut microbiota.

### 4.4 Disease characterization and pathogenesis

This category aims to understand and study the characteristics and pathomechanisms of cardiovascular disease. It may include Cluster #3 (cardiac remodeling), Cluster #6 (ventricular arrhythmia), and Cluster #8 (vascular smooth muscle cell). Enumerated below are some of the mechanistic modes availed to comprehend and scrutinize these ailments.

Worldwide, cardiovascular maladies figure as a principal contributor to global mortality. The development of the disease involves multiple physiological mechanisms, including vascular endothelial cell dysfunction, inflammatory responses, thrombosis, and lipid metabolism disorders. These mechanisms ultimately lead to the onset and progression of disease states such as atherosclerosis, myocardial ischemia, and myocardial infarction.

Atherosclerosis, typified by arterial wall thickening, constitutes a multifaceted cardiovascular disorder (65). It is characterized by cholesterol deposition, inflammatory response, and thrombosis, which can lead to serious cardiovascular events such as angina pectoris, acute myocardial infarction, and sudden death. Atherosclerosis is a major cause of coronary artery disease and cerebrovascular disease. Although this disease may not show any symptoms in the early stages, the course of the disease can change significantly when complicated by thrombosis. The progression of this disease comprises five distinctive phases: inception of early lesions, plaque rupture, plaque healing, and eventual fibrocalcification (66, 67).

Typically, the etiology of cardiovascular diseases can be associated with the impairment of vascular endothelial cells. This can affect blood viscosity, blood flow dynamics, immune and inflammatory responses, and contribute to the formation of atherosclerosis (68). The pathological mechanism of atherosclerosis is mainly due to impaired endothelial function, inflammatory response, and disrupted lipid metabolism. Accumulation of cholesterol and fatty acids within the vessel wall, facilitated by a compromised endothelium, engenders atheromatous plaques that incite an inflammatory reaction and enlist leukocytes. The leukocytes then secrete cytokines that exacerbate the endothelial cell damage and inflammatory response, creating a vicious cycle (69–71).

Atherosclerosis is associated with various risk factors, including hypertension, dyslipidemia, and diabetes, as well as poor lifestyle habits (72, 73). These risk factors contribute to the formation and development of atherosclerotic plaques, which can lead to the onset and progression of the disease (74). Moreover, inherent genetic predispositions may also factor in the pathogenesis of atherosclerosis in certain individuals. Retention of subendothelial lipoproteins is also a significant factor in the development of atherosclerosis (75).

Atherosclerosis is an inflammatory disease that has shown promise in therapies targeting cardiovascular inflammation (76, 77), such as drug development (78–80). Emerging novel therapeutic strategies, encompassing anti-atherosclerotic vaccines and pinpointed lipid-lowering therapies (81, 82), are being harnessed to augment the efficacy of established atherosclerosis treatments. These include cholesterol lowering, lifestyle changes, and the use of anti-atherosclerotic drugs (83).

### 4.5 TCM theory and symptom-specific treatments

This category devotes its attention to the deployment of Chinese herbal remedies tailored for specific symptoms or pathologies, encompassing blood stasis syndrome and cardiovascular diseases. It also covers the treatment of diseases through TCM theories, including Cluster #5 (blood stasis syndrome), Cluster #12 (metabonomic analysis), and Cluster #13 (positive feedback loop).

Coronary heart disease is predominantly acknowledged as an etiological agent of blood stasis syndrome (45). In line with the established theoretical framework of Chinese medicine, the pathological condition known as “blood stasis evidence” fundamentally manifests through impaired blood circulation, blood coagulation, and subsequent solidification culminating in the creation of blood stasis. This condition presents with specific clinical features, such as dark complexion, scaly and dry skin, cyanotic lips and nails, a purple or dark tongue, purple spots, tingling fixed in a certain area, and petechiae (47, 84, 85).

Within the TCM framework, the term “evidence” extrapolates a condensed and theorized abstraction of disease symptoms, in addition to an assemblage of clinical presentations and manifestations (86). It serves as the fundamental concept for diagnosis and treatment. TCM uses this concept to understand the internal homeostasis of the human body and guide the clinical application of herbal medicine.

Viewed through the lens of systems biology, “evidence” may signal aberrations within the protein network and gene regulatory network under perturbations (87). In therapeutic practice, Chinese medicine advocates treatment based on “zhen” as a basis. Consequently, divergent treatments are pursued if patients with the same disease present different “zhen”. Conversely, if the “zhen” are the same in different diseases, the treatment should be the same (84).

A profound linkage exists between atherosclerosis and blood stasis, the latter being a cardinal pathology precipitating the onset of atherosclerosis. Blood stasis is characterized by the obstruction of blood flow, increased blood viscosity, poor circulation, and possible coagulation of blood clots (88). This pathology is consistent with the platelet dysfunction observed in atherosclerosis, which leads to the formation of blood clots and poor local circulation (89). Contributory agents to this process may also encompass inflammatory predicators and free radicals (90).

Further, utilizing a biomedical research perspective reveals a noteworthy correlation between the presentation of blood stasis syndrome symptoms in coronary artery disease patients and critical parameters like disease severity, arterial disease intricacy, and stenosis extent. This correlation further supports the association between blood stasis syndrome and atherosclerosis (91).

Employing traditional Chinese remedies known for activating blood stasis, for instance, Tao Hong Si Wu Tang, and Qi Tonifying and Tongluo Granules, enables researchers to effectively alleviate alterations in hemodynamics and blood rheology, mitigate platelet activation and aggregation, and notably enhance blood circulation. By using traditional Chinese medicines that activate blood stasis, such as Tao Hong Si Wu Tang, and Qi Tonifying and Tongluo Granules, researchers can effectively improve hemodynamics and blood rheology, reduce platelet activation and aggregation, and enhance blood circulation (92–95). This can help prevent and treat atherosclerosis. Additionally, traditional Chinese medicine is capable of modulating the intestinal microbiome and thereby inducing alterations in plasma metabolites as a means of stasis treatment (96).

This intimates an interchangeably influential correlation between atherosclerosis and the blood stasis syndrome. The blood stasis syndrome pathology denoted in TCM theory illuminates fresh perspectives and elucidates novel methodologies for the comprehension and treatment of atherosclerosis.

### 4.6 Herbal treatment and its potential in complementing

This division explores numerous scholarly investigations that unfold the synergistic enhancement of conventional therapies for atherosclerotic disease with the use of herbs and Traditional Chinese Medicine (TCM). It encompasses assorted thematic clusters like the therapeutic potential, myocardial ischemia-reperfusion injury, coronary heart disease, tongxinluo capsule, action on the cardiovascular system, and the utilization of golden herbal medicine.

#### 4.6.1 Improvement of vascular endothelial function

Sustaining the vascular endothelial function within normal parameters is instrumental for the health of multiple organs and establishing homeostatic balance in the body (97). Further, endothelial dysfunction is associated with the initiation and progression of various cardiovascular and cerebrovascular maladies (98).

Many studies have shown that drugs and herbs improve vascular endothelial function by modulating oxidative stress, anti-inflammatory processes, and altering endothelial cell behavior. For example, tetramethylpyrazine (TMP) and vanillic acid from Ligusticum chuanxiong have a variety of pharmacological effects, including antioxidant, anti-inflammatory, and anti-apoptotic effects, and modulate vasodilation and endothelial protection, among others (99, 100). Additionally, tanshinone B, ferulic acid, and astragalin along with its derivatives exert direct influence on vascular endothelial cell function, stimulate angiogenesis, and showcase cardioprotective capabilities (101, 102).

Specific mixtures of herbs, notably Ge Gen Baicalin Wan and Sheng Wei San, possess the capacity to impart vasoprotective effects by orchestrating the modulation of distinct signaling cascades, including the HMGB1/NF-κB/NLRP3 and ROCK/cofilin pathways (103, 104). Additionally, Xinkeshu, through its enhancement of endothelial progenitor cell-facilitated endothelial regeneration, and rhodopsin, by modulating the hepatic low-density lipoprotein receptor (LDLR) and endothelial nitric oxide synthase (p-eNOS), potentially contribute positively to the improvement of endothelial functionality (105, 106).

Moreover, herbal formulations, namely Shengmusan, Xinkeshu, and Qiliqiangxin capsule, could aid in enhancing endothelial functionality in pathologies like blood-brain barrier dysfunction and coronary artery disease, as well as in the mediatory role of Piezo1 channels in reducing blood-brain barrier permeability, thereby improving cardiovascular disease conditions (107, 108).

#### 4.6.2 Reducing the inflammatory response

The role of chronic inflammation in the manifestation of cardiovascular disease is widely acknowledged, principally due to its associated acceleration of atherosclerosis, destabilization of plaque formations, impairment of endothelial functionality, and consequent propagation of atherosclerosis (109). Therefore, inhibition of the inflammatory response has become an important component of many treatments.

Numerous pharmacological agents have shown promising results in impeding the inflammatory response; a primary example is the Li Y. Qing-Xue-Xiao-Zhi formula, which has demonstrated therapeutic efficacy on atherosclerosis by advancing lipid efflux and suppressing the macrophage-mediated inflammatory reaction (110). Further, Salvinorin exhibits potential effectiveness in both the prevention and treatment of initial and progressive atherosclerotic stages by attenuating inflammatory responses (111). Additionally, both rhodopsin and rhodopsinic acid could potentially mitigate inflammation by diminishing the expression of inflammatory contributors in macrophages. For example, *in vitro* experiments found that in LPS-induced RAW264.7 cells, rhodopsin (10, 20, and 40 μM) for 18 h significantly reduced the expression of inflammatory factors such as TNF-α, IL-1β, and IL-6 (112). Chang WC found that rhodopsinic acid (1–10 μg/ml, effective for 24 h) inhibited inflammatory factors such as TNF-α, IL-1β, and IL-6 in ATP-treated THP-1 cells by reducing the expression of IL-1β, TNF-α, and NLRP3 inflammasome levels in ATP-treated THP-1 cells to inhibit M1 macrophage activation (113). And rhubarbic acid (0–35 μm, 26 h) significantly reduced M1 macrophage-associated pro-inflammatory cytokines by inhibiting LPS-induced expression of NF-κb and IκB kinase β (IKKβ) in RAW 264.7 cells (114). Concurrently, Dendrobine and Xiaoyaosan have been discovered to impede intracellular inflammatory responses and oxidative stress. Specifically, Dendrobine is capable of curtailing inflammatory reactions and oxidative stress via the avenue of FK506-binding protein 1A-associated autophagy ox-LDL in human umbilical vein endothelial cells subjected to Dendrobine treatment (115). Through ex vivo experiments, Chen M uncovered that Xiaoyaosan effectively hampers the ox-LDL-induced production of inflammatory agents in macrophages (116).

Furthermore, certain therapeutic approaches stifle inflammation by obstructing specific biological pathways, for instance, through the use of triptolide, Astragalus membranaceus, maslinic acid, total flavonoids from Engelhardia roxburghiana wall, and sodium danshensu—all of which repress inflammation via inhibiting the NF-κB pathway (117–121), modified yuejuwan inhibits cholesterol accumulation and inflammation in THP-1 macrophage-derived foam cells by inhibiting the activity of the TRIM37/TRAF2/NF-κB pathway (122), and 13-methylberberine inhibits NLRP3 inflammasome activation by inducing autophagy in human umbilical vein endothelial cells (123), Further, paeonol demonstrates its capacity to retard the progression of inflammation by suppressing the downstream NLRP3 inflammasome through the upregulation of miR-223 levels in plasma-derived exosomes in hyperlipidemic rat models (124).

Furthermore, recent studies have also identified the important role of inflammatory cytokines such as IL-1, IL-18, and IL-33 in the inflammatory process, which have emerged as novel targets to inhibit the activity of NLRP3 inflammatory vesicles and thus inhibit the inflammatory response (119, 125). Research studies have evidenced Shenlian's potential to curtail the secretion of inflammatory cytokines such as interleukin-1 (IL-1), interleukin-18 (IL-18), and interleukin-33 (IL-33), along with its ability to impede the activity of NLRP3 inflammatory vesicles (126). The succinate/HIF-1α/IL-1β inflammatory signaling axis (succinate axis) is capable of promoting atherosclerotic inflammatory plaque progression, and Xu J demonstrated that Pue-Tan prevented atherosclerotic inflammatory plaque formation and slowed the pathological process of AS by targeting HIF-1α to block the succinate axis (127). Additionally, puerarin manifested capability in thwarting the progression of cardiovascular diseases by enhancing mitochondrial functionality and quelling the inflammatory response (128).

#### 4.6.3 Regulation of lipid metabolism

There is a close association between atherosclerosis and the dysregulation of lipid metabolism (129). Dysregulation of lipid metabolism often manifests as abnormal lipid levels and is therefore considered one of the most common diagnostic indicators of AS (130). The pathogenesis of AS is markedly influenced by alterations in lipid metabolism. Lipids in plasma exist as lipoproteins and undergo a variety of chemical modifications, such as defatting and oxidation, which penetrate the arterial wall and lead to lipid deposition, forming the initial stages of atherosclerosis (131). Therefore, regulating lipid metabolism may be one of the effective ways to prevent and treat atherosclerosis.

Autophagy, a biological process regulated by autophagy-related genes, enables eukaryotic cells to use lysosomes to degrade their own cellular content and pre-existing damaged organelles (132). Recent academic endeavors have investigated the potential therapeutic interventions for cardiovascular and cerebrovascular diseases by modulating biological processes like lipid metabolism and autophagy, with a keen focus on utilizing traditional Chinese medicines and natural products. These explorations have yielded promising new therapeutic strategies. For example, Tong-Qiao-Huo-Xue decoction can inhibit autophagy and resist ischemic injury by regulating the PI3K/Akt/mTOR target pathway (133). celastrol inhibits lipid accumulation through the LXR*α*/ABCA1 signaling pathway and autophagy in vascular smooth muscle cells (134). The same S. baicalensis may play a therapeutic role by modulating cholesterol biosynthesis and sphingolipid metabolic pathways (135). In addition, rhubarb and gypenoside, a butanol extract of Acanthopanax senticosus, have been studied and shown to have significant lipid-lowering effects (136–138). Certain herbal formulations, including Dingxin Recipe IV, DiDang decoction, Yindanxinnaotong, and Shengmai Yin, exhibit potential efficacy in enhancing lipid metabolism and providing resistance to atherosclerosis (136–139) (139–142).

Macrophages, pivotal in lipid metabolism and capable of phagocytosing lipids, dead cells, and other perceived threats, present a potential target for therapeutic regulation in the treatment of atherosclerosis (143). For instance, ginsenoside Rb1 exhibits the ability to modulate macrophage polarization, thereby mitigating atherosclerosis (144), Araloside C manipulates macrophage polarization via Sirt1-mediated autophagy, consequently lessening atherosclerosis (145), and danshensu promotes cholesterol efflux from RAW264. 7 macrophages and reduced intracellular lipid accumulation (146). Additionally, oridonin escalates the protein content relevant to lipid efflux whilst reducing that of lipid uptake within macrophages (147). In addition, Jieduquyuziyin prescription was also effective in alleviating atherosclerosis symptoms, mainly by regulating cholesterol efflux and inhibiting TLR9/MyD88 activation (148). The QiShenYiQi pill hinders atherosclerosis by stimulating cholesterol retrogradation through the PPARγ-LXRα/β-ABCA1 pathway, consequently diminishing lipid deposition (149).

#### 4.6.4 Reduction of foam cells

The genesis and accumulation of foam cells constitute a pivotal phase in the development and advancement of atherosclerosis (150). Their formation is largely determined by the balancing action of three major interrelated biological processes, including lipid uptake, cholesterol esterification, and cholesterol efflux (151). In the process of atherosclerosis progression, lipids incrementally build up in the subendothelial space of impaired arteries. These lipids are subsequently assimilated by macrophages within the arterial wall, thereby leading to an excessive accumulation of cholesterol esters and subsequent foam cell development (152). The persistence of cholesterol-filled foam cells in the arterial wall further promotes the development of atherosclerosis (153). Diverse natural products and pharmaceuticals may hold significant promise in counteracting foam cell formation and the subsequent progression of atherosclerosis by interfering with pertinent biological processes.

Certain pharmaceuticals have the potential to minimize foam cell formation or accumulation by inhibiting the uptake of ox-LDL. For example, Cai Y found that Huang-Lian-Jie-Du soup inhibited ox-LDL-induced foaminess in RAW264.7 macrophages in *in vitro* experiments (154). Tans Hindiol C inhibited oxidized LDL-induced macrophage foam cell formation via a peroxiredoxin 1-dependent pathway (155). Substances such as geniposide, gentiana, and celosins exhibit a capacity to diminish macrophage phagocytosis of lipids (156–158).

Moreover, compounds such as gypenoside, nicotinate-curcumin, and calycosin, derived from Radix Astragali, impede foam cell formation by augmenting autophagy (159, 160). Concurrently, diterpenoids endorse macrophage cholesterol efflux through the orchestration of the PPARγ-LXRα-ABCA1 pathway (161), curcumin promotes cholesterol efflux from THP-1 macrophages through the miR-125a-5p/SIRT6 axis (162); and paeonol inhibits cholesterol efflux from THP-1 macrophages by enhancing LXRα-ABCA1-dependent cholesterol efflux to inhibit ox-LDL-induced macrophage foam cell formation (163).

Certain pharmaceuticals inhibit foam cell formation by modulating various signaling pathways. For example, research has shown that LongShengZhi capsules reduce foam cell accumulation by upregulating the expression of ABCA1/ABCG1 (164). Three polymethoxyflavones from the peel of Citrus reticulata “Chachi” inhibited lipid uptake by downregulating SRA1/CD36 expression and promoted lipid uptake by upregulating PPARγ/LXRα/ABCG1/SRB1 expression. reticulata “Chachi” inhibited lipid uptake by downregulating SRA1/CD36 expression and promoted cholesterol efflux from foam cells by upregulating PPARγ/LXRα/ABCG1/SRB1 expression (165). Fu Fang Dan Shen blocked LPS-induced monocyte activation and foam cell formation by inhibiting NF-κB (166). The Dan-Lou formula deterred ox-LDL-induced foam cell formation operating via the TLR4/NF-κB and PPARγ pathways (167). *In vitro* experiments showed that Xinmaikang reduced foam cell formation by modulating the PINK1/Parkin pathway, and Naoxintong inhibited foam cell formation by activating the Ppar*α* pathway (168). Shen-Yuan-Dan capsules ameliorated atherosclerosis in conjunction with foam cell formation, courtesy of the fortification of autophagy and suppression of the PI3K/Akt/mTORC1 signaling pathway—both actions thereby diminishing atherosclerosis and foam cell formation (169). Danlou Tablets promote macrophage autophagy by inhibiting the PI3K/Akt/mTOR signaling pathway to reduce foam cell formation and improve atherosclerosis (170). Furthermore, Li Y discovered that triterpenoids and polysaccharides from Ganoderma lucidum propelled foam cell apoptosis by ameliorating endothelial dysfunction and inflammatory polarization of macrophages (171).

#### 4.6.5 Regulation of gut microbes and their metabolites

The imbalance or dysbiosis of gut microbiota serves as a crucial pathogenic determinant in the onset of dyslipidemia (172). Both the gut microbiota and its metabolic product—trimethylamine-N-oxide (TMAO)—have been robustly associated with atherosclerotic development (173). In addition, the gut flora promotes the progression of atherosclerosis by interfering with the normal metabolism of bile acids (174). Consequently, the gut microbiota holds significant influence in the initial development and subsequent progression of atherosclerosis. Mitigating atherosclerosis may entail modulation of the gut microbiota, alteration of its metabolic activity, reshaping of its structural traits, among other strategies.

Tetrahydroxy-stilbene-2-O-β-D-glucoside mitigates atherosclerosis in ApoE^−/−^ mice, leveraging the modulation of intestinal microbiota (175). Qing-Xin-Jie-Yu granules attenuate atherosclerosis by remodeling the intestinal microbiota and metabolic homeostasis in ApoE^−/−^ mice (176). Guanxinning tablets attenuated coronary atherosclerosis by modulating the gut microbiota and its metabolites in Tibetan piglets induced by a high-fat diet (177). Petitgrain addresses atherosclerosis by suppressing the production pathway of choline-TMA-TMAO in the gut microbiota, leveraging its vitamin-like properties (178, 179). Ginkgo biloba extract ameliorates atherosclerosis by reinstating equilibrium in gut microbiota and their metabolic processes (180).

In therapeutic practice, a common strategy is the modulation of lipid metabolism pathways in gut microbiota. As an instance, Zhang Y demonstrated that Dingxin Recipe IV mitigated atherosclerosis by adjusting lipid metabolism via the LXR-α/SREBP1 pathway and manipulating the gut microbiota of ApoE^−/−^ mice fed a high-fat diet (141). Ferulic acid mitigated atherosclerosis-related injury by maneuvering gut microbiota dynamics and lipid metabolism (181). Banxia Xiexin soup mitigated atherosclerotic conditions by supervising the lipid metabolism axis of gut microbiota (182).

Furthermore, it also restrains the progression of atherosclerosis by managing the metabolism of bile acids. For example, HuangQi ChiFeng soup sustains gut microbiota and bile acid homeostasis via FXR signaling, thereby mitigating atherosclerosis (183). Resveratrol manages TMAO synthesis and bile acid metabolism by remodeling the gut microbiota composition, hence attenuating TMAO-induced atherosclerosis (184).

### 4.7 Challenges and opportunities

Given that a bulk of the research zeroes in on the potential influence of variant herbal remedies and therapeutic strategies on cardiovascular disease, there is a pressing need for additional clinical trials to solidify the efficacy and safety of these modalities. The assurance of quality and purity of the utilized herbs and formulations is essential, as it could influence the effectiveness of the treatment. Further exploration of the molecular mechanisms, especially those relevant to gut microbiota and the influence of herbal constituents on lipid metabolism, in the treatment of cardiovascular disease could also be warranted.

A multitude of studies centers on delving into the potential applications of herbal medicine in combatting cardiovascular diseases, signaling sizeable potential for the creation of innovative drugs and therapies. And according to the keyword citation explosion, the importance of aspects such as oxidized nitric oxide enzymes, vascular smooth muscle cells, restenosis, and integrative medicine in the treatment of cardiovascular diseases is being increasingly recognized, which may provide new directions and targets for future research. Continued investigations into traditional Chinese medicine interventions for cardiovascular disease could create avenues for the successful incorporation of some time-honored therapies into contemporary medicine. Corresponding studies have illustrated the potential in enhancing cardiovascular health through the modification of gut microbiota, insinuating the possibility of crafting novel therapies centered on this premise in the future.

### 4.8 Limitations of this study

This study undeniably exhibits certain intrinsic shortcomings and limitations. To begin with, this investigation solely incorporated the Web of Science (WOS) database, leaving out other prominent databases like Google Scholar, PubMed, and Embase. Consequently, the data at hand might not entirely represent the scope of traditional Chinese medicine (TCM) in relation to atherosclerosis research. Furthermore, owning to the confined search parameters, in terms of literature language and selection criteria for literature types, our dataset may not encompass all pertinent articles. This could result in our study not portraying the complete breadth of atherosclerosis-related disorders. Resultantly, there might be inherent bias introduced in the outcome analysis premised on the extant literature. Prospective studies should strive to circumnavigate these constraints as much as feasible, facilitating a more substantial and inclusive analysis.

## 5 Conclusion

In sum, we have furnished an initial depiction of the global landscape and tendencies of Traditional Chinese Medicine (TCM) research, grounded in bibliometric and visual analytic methodologies. Investigations in this domain keenly concentrate on employing TCM while integrating Western therapeutic ideologies for the management and prophylaxis of cardiovascular conditions. The scope of research generally ranges from the study of individual Chinese medicines, to the exploration of compounded Chinese medicines, to the investigation of disease mechanisms. Studies show that in recent years, attention in this field has focused on several aspects, including disease characteristics and pathomechanisms, TCM theories and treatment of characteristic signs and symptoms, the therapeutic potential of herbal medicines, and the effects of TCM on intestinal flora. Crucial mechanisms encompass overseeing endothelial function and restraining inflammatory responses, modulating lipid metabolism and curbing foam cell formation, and managing intestinal microbes and their metabolites. An important approach observed in these themes is the alleviation of atherosclerosis and its related problems by modulating the gut microbiota. Evidence indicates that by adjusting the lipid metabolism axis of gut microbiota, controlling bile acid metabolism, and refining the structure of gut flora, TCM could offer innovative preventive and therapeutic strategies against atherosclerosis.

Overall, the field aims to explore and validate the potential of new or established TCM in the prevention and treatment of cardiovascular diseases, with the goal of providing more effective therapeutic approaches for the treatment of cardiovascular diseases. Simultaneously, there is a pronounced emphasis on probing and gaining an understanding of the disease’s pathogenesis to equip physicians with better strategies for disease prevention and management.

## Data Availability

The original contributions presented in the study are included in the article/**Supplementary Material**, further inquiries can be directed to the corresponding author.
